# SB4 in the Era of Biosimilars: Clinical Data and Real-World Experience

**DOI:** 10.31138/mjr.30.1.59

**Published:** 2019-05-31

**Authors:** Shing T. Law, Peter C. Taylor

**Affiliations:** Botnar Research Centre, Nuffield Department of Orthopaedics, Rheumatology and Musculoskeletal Sciences, University of Oxford, Oxford, United Kingdom

**Keywords:** Biosimilar, anti-TNF, SB4; rheumatoid arthritis, psoriatic arthritis, ankylosing spondylitis, real-world data

## Abstract

A biosimilar is a biological medicinal product that is highly similar to an already authorized original biological medicinal product. The introduction of biosimilars may allow for a reduction in health care costs, due to discount pricing. Current clinical studies and real-world data suggest that the biosimilar SB4 is equivalent to etanercept with respect to efficacy and safety. Additional real-world safety data for SB4 via pharmacovigilance studies are needed to draw conclusions regarding the risks of rare adverse events such as serious infections and malignancy. Clinical trial design of biosimilars should be standardised to improve consistency, increase confidence and facilitate interpretation of data. Where there are health economic advantages of switching from originator to biosimilar, patients should be appropriately informed, and, ideally, in order to minimise nocebo responses and maximise benefit, switching should be undertaken by shared decision-making between the physician and patient on a case-by-case basis.

## INTRODUCTION

A biosimilar is a biological medicinal product that is highly similar to an already authorized original biological medicinal product (reference medicinal product) in terms of quality, safety and efficacy, based on a comprehensive comparability exercise (U.S. Department of Health and Human Services, Food and Drug Administration, Centre for Drug Evaluation and Research [CDER], Centre for Biologics Evaluation and Research [CBER, 2015], Scientific Considerations in Demonstrating Biosimilarity to a Reference Product Guidance for Industry, European Medicines Agency [2014] Guideline on similar biological medicinal products containing biotechnology-derived proteins as active substance: non-clinical and clinical issues). The introduction of biosimilars may allow for a reduction in health care costs, due to discount pricing.^1^ Clinical data and real-world evidence are needed to support uptake of biosimilars in clinical practice.

Etanercept (ETN; Enbrel^®^, Pfizer, New York, NY, USA) is a recombinant human tumour necrosis factor (TNF) receptor (TNFR) p75Fc fusion protein.^2^ The etanercept bio-originator was the first TNFi to gain approval from the United States Food & Drug Administration (FDA) and the European Medicines Agency (EMA) for the treatment of moderate to severe rheumatoid arthritis in 1998.^3^ Additional licenced indications for etanercept include plaque psoriasis, psoriatic arthritis, ankylosing spondylitis, axial spondylarthritis and polyarticular juvenile idiopathic arthritis.^3^ The recent patent expiration of the etanercept originator ETN in 2015 in Europe has facilitated the development of biosimilar products. In the US, the patent of etanercept has been extended until 2028.^4^

A biosimilar of etanercept is SB4 (Benepali, Samsung Bioepis UK Limited, Surrey, UK; Brenzys, Samsung Bioepis, Republic of Korea). SB4 received EU marketing authorisation in January 2016. This authorisation was based on the totality of evidence from pre-clinical and clinical Phase I and III studies that demonstrated similar efficacy, bioequivalence, and comparable safety and immunogenicity to ETN. In a phase I, randomised, single-blind, three-part, crossover study of etanercept and SB4, their pharmacokinetics were equivalent in healthy male subjects.^5^

Optimal trial designs of biosimilar switching studies have been discussed previously.^6^ There is limited clinical and real-world evidence on the outcomes of transition from ETN to SB4 in routine clinical practice regarding efficacy, safety, and acceptability to patients. In a phase III double-blind study, 596 patients with active rheumatoid arthritis despite methotrexate therapy were randomized to receive etanercept or SB4.^7^ The primary endpoint was the American College of Rheumatology (ACR) measure of 20% improvement (ACR20) with an equivalence margin of −15% to 15% in line with EMA guidelines (Committee for Medicinal Products for Human Use [CHMP, 2005] Guideline on the choice of the non-inferiority margin, http://www.emea.eu.int). The proportion of patients meeting ACR20 in the per-protocol set was 78.1% for SB4 and 80.3% for etanercept at week 24 (95% CI of the adjusted treatment difference was −9.41% to 4.98%), and 80.8% for SB4 and 81.5% for etanercept at week 52. 165 (55.2%) patients in SB4 and 173 (58.2%) patients in ETN reported at least one treatment-emergent adverse event.

Of 245 patients entering the open-label extension study to week 100, 126 continued to receive SB4 (SB4/SB4) and 119 switched to SB4 (ETN/SB4).^8^ ACR response rates were sustained and comparable between SB4/SB4 and ETN/SB4 with ACR20 response rates at week 100 of 77.9% and 79.1%, respectively. Other efficacy results, including radiographic progression, were also comparable between the groups. After week 52, rates of treatment-emergent adverse events were 47.6% (SB4/SB4) and 48.7% (ETN/SB4).

Switching from etanercept to SB4 did not result in any increase in immunogenicity.^8^ There were no statistically significant differences in rates of adverse events between groups other than for injection site reactions: 3.7% in the SB4 group compared to 17.5% in the ETN group at week 52 (p < 0.001). The incidence of anti-drug antibodies (ADAs) was significantly lower in the SB4 group at week 52 (0.7% compared to 13.1%). The additional ADAs detected to etanercept were transient, of low titre and detected mostly at early time points between weeks 4 and 8. ADAs largely did not interfere with the activity of ETN, indeed only one ETN patient developed ADA titres of neutralising capacity. The EMA felt that the differences in ADA incidence between etanercept and SB4 may have been due to different drug concentrations in samples or different analytical methods. Moreover, the EMA concluded that the observed differences with respect to ADA formation appeared to be transient, with almost no differences after 8 weeks of treatment, therefore their clinical significance was considered minimal (Committee for Medicinal Products for Human Use. Assessment report: Benepali. 2015. http://www.ema.europa.eu/docs/en_GB/document_library/EPAR_-_Public_assessment_report/human/004007/WC500200380.pdf). Higher-level hepatobiliary disorders were reported with SB4 compared with etanercept (17 vs. 0 adverse events), all reported pre-switch, was thought to be due to chance rather than to true SB4 causality.^6,9–11^ Difference between SB4 and reference etanercept hepatobiliary disorders response to letter). There were four malignancies in the SB4 group compared to one in the ETN group, but the EMA concluded that these numbers were too low to conclude on significance (Committee for Medicinal Products for Human Use [CHMP], European Medicines Agency [2015] Benepali).

In 2016, Tweehuysen et al. undertook a six month open-label controlled cohort study of non-mandatory transition from etanercept to SB4 in patients with an inflammatory rheumatic disease.^12^ A key difference between blinded and open-label transitioning is the level of awareness of the transition by patients and physicians, which may induce nocebo effects when transitioning to a biosimilar (see article in series). Of 642 ETN-treated patients, 635 (99%) agreed to transition from originator ETN to biosimilar SB4, of whom 625 patients (433 with rheumatoid arthritis, 128 with psoriatic arthritis, and 64 with ankylosing spondylitis) were included in the transition cohort, and 600 ETN-treated patients from 2014 were included in the historical cohort. The study showed a near optimal treatment acceptance rate of 99% and treatment persistence rate of 90% at month 6. Non-mandatory transitioning from ETN to SB4 using a specifically designed communication strategy resulted in a slightly lower 6-month treatment persistence rate and smaller decreases in disease activity in the transition cohort compared to the historical cohort. However, where these differences are clinically relevant are debatable because of potential nocebo effects, and potential different clinical practices between the transition cohort and historical cohort.

Short term real-world data from the British Society for Rheumatology Biologics Registers for RA (BSRBR-RA) demonstrated comparable short-term effectiveness of SB4 with ETN in terms of drug response, drug survival and safety profile.^13^ Of 322 and 855 patients starting ETN or SB4 respectively as first biologic recruited to the BSRBR-RA, the adjusted hazard ratio for stopping ETN versus SB4 at their first follow up was 1.0 (95% CI 0.4–2.5, *p* = 0.9). The risk of serious adverse event over the first six months was also similar between the SB4 and ETN groups (hazard ratio = 0.5, 95% CI 0.3–1.1, *p* = 0.1), with 13 (14%) and 19 (6%) serious adverse events reported in ETN and SB4 patients respectively until first follow up. In UK, etanercept biosimilars are now frequently used as first-line biologics in rheumatoid arthritis patients.

In the SMaRT study, 92 patients with rheumatoid arthritis, psoriatic arthritis and ankylosing spondylitis who switched from Etanercept to SB4 were followed for more than six months.^14^ After six months following the switch, 91% of patients using SB4 have continued. Following the switch, 8 patients (9%) stopped SB4. The reasons for this were 7/8 clinician/patient determined inefficacy (6 returned to etanercept, 1 switched to certolizumab), 1/8 switched after reporting palpitations and poor concentration.

Of 147 patients on etanercept in Sweden, switching to SB4 in a real-life setting was acceptable to most patients.^15^ Low mean disease activity was also maintained in the rheumatoid arthritis and psoriatic arthritis groups. The Benefit Study is a pan-European observational study to evaluate real-world effectiveness of SB4 following transition from originator etanercept in patients with rheumatoid arthritis or axial spondyloarthritis.^16^ Interim analysis of the Benefit study showed in 255 subjects (study designed to enrol 600 subjects), the mean individual change in disease score from baseline to 3 months post-transition were 0.0 (95% CI −0.1, 0.2) and 0.4 (95% CI 0.0, 0.9) in rheumatoid arthritis and axial spondylarthritis subjects respectively. Additional 3- and 6-month outcomes are forthcoming (https://clinicaltrials.gov/ct2/show/NCT03100734).

Current clinical studies and real-world data suggest that SB4 is equivalent to etanercept in efficacy and safety. Additional real-world efficacy and safety data of SB4 via pharmacovigilance studies are needed to draw conclusions regarding benefits and risks of adverse events such as serious infections and malignancy. Clinical trial design of biosimilars should be standardised to improve consistency and increase confidence.^17^ Switching should remain a case-by-case clinical decision by the physician and patient.
